# Topological design of strain sensing nanocomposites

**DOI:** 10.1038/s41598-022-13393-w

**Published:** 2022-06-02

**Authors:** Long Wang, Wei-Hung Chiang, Kenneth J. Loh

**Affiliations:** 1 Department of Structural Engineering, University of California San Diego, La Jolla, CA 92093-0085 USA; 2 Department of Civil and Environmental Engineering, California Polytechnic State University, San Luis Obispo, CA 93407 USA; 3 Department of Chemical Engineering, National Taiwan University of Science and Technology, Taipei, 106 Taiwan

**Keywords:** Mechanical engineering, Sensors and biosensors

## Abstract

High-performance piezoresistive nanocomposites have attracted extensive attention because of their significant potential as next-generation sensing devices for a broad range of applications, such as monitoring structural integrity and human performance. While various piezoresistive nanocomposites have been successfully developed using different material compositions and manufacturing techniques, current development procedures typically involve empirical trial and error that can be laborious, inefficient, and, most importantly, unpredictable. Therefore, this paper proposed and validated a topological design-based methodology to strategically manipulate the piezoresistive effect of nanocomposites to achieve a wide range of strain sensitivities without changing the material system. In particular, patterned nanocomposite thin films with stress-concentrating and stress-releasing topologies were designed. The strain sensing properties of the different topology nanocomposites were characterized and compared via electromechanical experiments. Those results were compared to both linear and nonlinear piezoresistive material model numerical simulations. Both the experimental and simulation results indicated that the stress-concentrating topologies could enhance strain sensitivity, whereas the stress-releasing topologies could significantly suppress bulk film piezoresistivity.

## Introduction

Polymer nanocomposites typically comprise of conductive or semi-conductive nanofillers (e.g., carbon nanotubes (CNTs), graphene, and metal nanoparticles and nanowires) dispersed in nonconductive polymer matrices. There has been growing research interests in nanocomposites mainly since these materials possess superior and unique electrical, thermal, mechanical, optoelectronic, and chemical properties^1–3^. In addition, inherent to the nanofillers is their large surface area-to-volume ratio, where these surfaces can be functionalized with different molecular species for tuning their functional properties that lead to enhanced macro-scale material properties^4–6^.

Among the various unique properties of nanocomposites, piezoresistivity is commonly observed in various nanocomposite material systems^7–11^. Their piezoresistivity typically stems from three primary mechanisms, namely, the nanofillers’ intrinsic piezoresistivity, tunneling effect, and contact resistance of the nanofiller networks^10,12^. Unlike conventional foil-based strain gages and semiconducting materials (e.g., doped silicon) that are intrinsically rigid and non-stretchable, high-performance nanocomposites possess superior mechanical robustness, flexible properties, and high strain sensitivities^5,10,13^. In fact, piezoresistive nanocomposites have demonstrated their potential for measuring strains in various types of structural systems, including engineered structures^11,14,15^ and biological systems^7,10,12,16,17^.

On the other hand, the sensitivity to applied strains can be undesirable in certain circumstances. For example, stretchable conductors are promising candidates for flexible displays^18^, flexible energy harvesting and storage^19^, and artificial skins^10,20^, among others. It remains challenging to develop highly flexible conductive materials whose electrical conductivity remains constant even during large deformations (e.g., stretching, bending, and twisting). In addition, for the next-generation of wearable electronics, it is essential to develop flexible sensing materials that provide stable electrical outputs related to multiple parameters (e.g., temperature and pH) without being affected by applied strains and loads^21,22^. Therefore, there remains a need to effectively design the piezoresistive properties of functional materials for different target applications. To be specific, these functional materials may require enhanced piezoresistivity for operation as a highly sensitive strain sensor or, in a different scenario, exhibit suppressed piezoresistivity to prevent strain- or load-induced effects that can damage sensitive components.

To develop functional nanocomposites with desirable piezoresistive performance, most of the reported work to date focused on engineering and enhancing the material aspect of the nanocomposites, such as the nanostructures of the nanofillers^21,23–25^, nanofiller loading or concentrations^26^, and morphologies of the polymeric matrices^10,27^, to name a few. Although these strategies were successful at adjusting nanocomposite piezoresistivity, the processes used were mostly empirical and inefficient, especially considering the complex effects of the resulting material systems on global piezoresistive behavior. More importantly, the potential multifunctionality of the nanocomposites could hardly be leveraged given that each material system had to be developed for a specific target application. Thus, it is essential to develop a more universal materials engineering methodology that is not only broadly applicable to different nanocomposites but also capable of achieving a wide range of desired piezoresistivity depending on the application needs.

Therefore, this paper proposes and validates a topological design-based approach to strategically control the strain sensing performance of nanocomposites. The hypothesis is that bulk strain sensitivity can be manipulated and controlled by patterning the nanocomposite to form topologies that modify its external load-induced stress field distribution. First, to test this hypothesis, hierarchical inhomogeneous structures were designed to concentrate stresses, while Kirigami cut structures were implemented to release stresses for enhancing and suppressing strain sensitivity, respectively. Second, finite element (FE) numerical simulations were conducted to verify the effects of different topologies on their corresponding stress field distributions. Third, based on the simulation results, two types of patterned thin films were reproduced experimentally (i.e., by screen-printing graphene nanosheet (GNS)-ethyl cellulose (EC) and spray-coating CNT-latex thin films) onto laser-cut polyethylene terephthalate (PET) substrates. They were then subjected to load tests for characterizing their strain sensitivity and for validating the effectiveness of topological design on controlling bulk film strain sensitivity. Last, linear and nonlinear material models were developed to establish a method for designing patterned materials with specific piezoresistive properties. The models’ electromechanical properties were compared with experimental test results to demonstrate their validity.

## Results and discussion

### Topological designs

**Figure 1:**
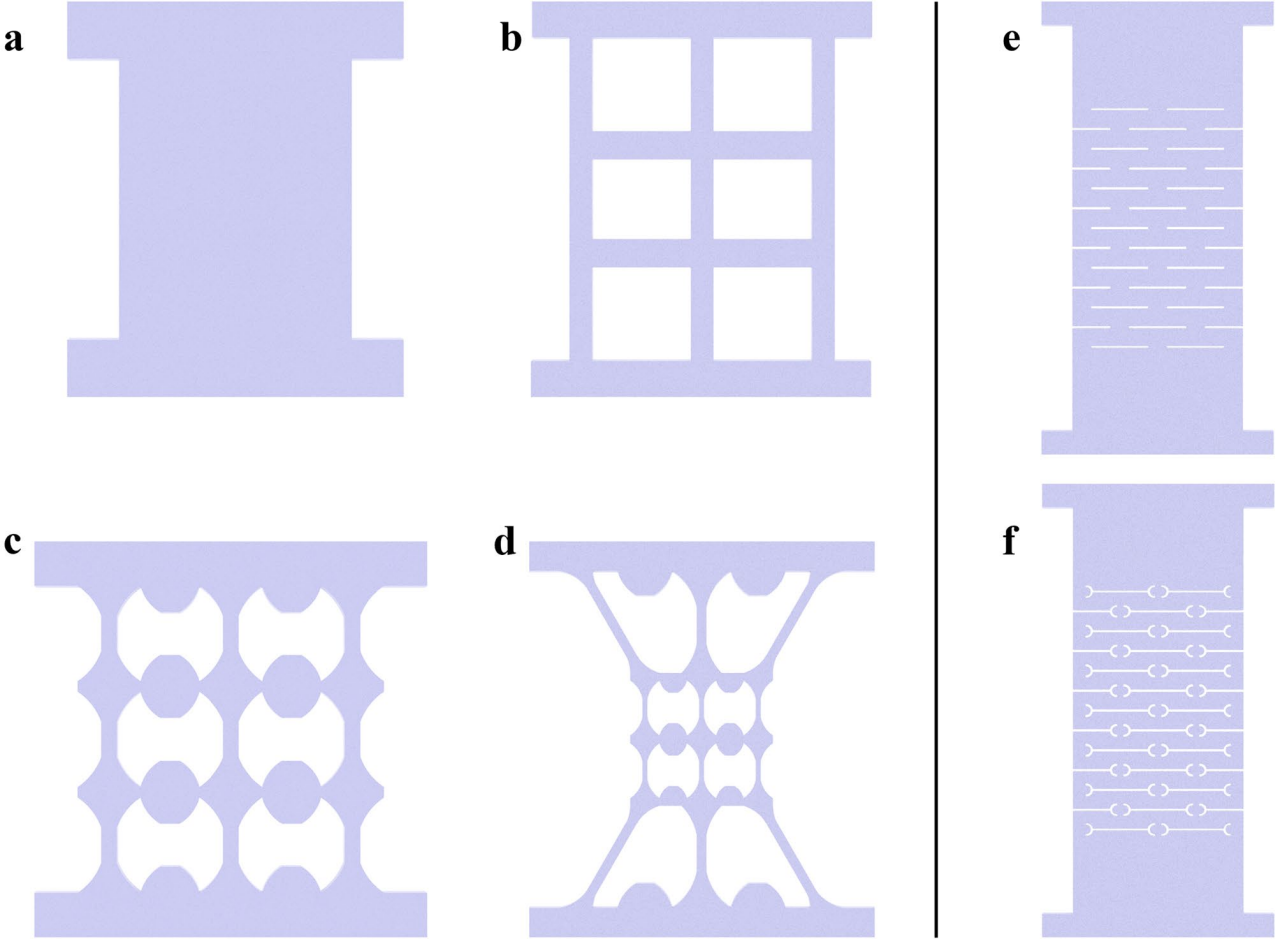
Different topological designs. (**a**) Non-patterned, (**b**) Grid, (**c**) Dog-Bone Grid, (**d**) Hierarchical Dog-Bone, (**e**) Kirigami, and (**f**) Modified Kirigami topologies.

Many thin film resistive strain sensors are based on a continuous, rectangular geometry, which was employed as the Non-Patterned control set in this study (Fig. 1a). Since the aim of this study was to manipulate thin film strain sensing properties when the material system remained unchanged, different topological designs were proposed. By varying thin film topologies, the tension-induced stress and strain distributions in the patterned material system could be altered and controlled. Here, two main categories of different topologies were designed, namely, stress-concentrating and stress-releasing topologies, which are presented in Fig. 1b–d,e,f, respectively.

First, the stress-concentrating designs started with the creation of a grid-like pattern in lieu of a continuous rectangular geometry (i.e., the Grid as shown in Fig. 1b and Supplementary Fig. 1a). Second, the introduction of stress concentrations was based on the inhomogeneous stress distribution in the commonly used dog-bone test coupon. It is well-known that a dog-bone-shaped structure pulled in tension would result in concentrated stresses and strains in its tapered center region. Thus, the vertical elements of the grid design in Fig. 1b were substituted with dog-bone elements to purposely introduce inhomogeneity to the structure (i.e., considering that tension is applied along the vertical direction), as shown in Fig. 1c (i.e., the Dog-Bone). The detailed dimensions of the dog-bone unit are illustrated in Supplementary Fig. 1b,c. In addition, the horizontal elements were replaced with an inverse dog-bone shape to combat Poisson’s effect. Last, to further enhance stress concentrations, a hierarchical design was employed, where the shape of the entire grid was modeled after a dog-bone structure (Fig. 1d and Supplementary Fig. 1d–f). This design also entailed the incorporation of smaller dog-bone units as the vertical elements, as is shown in Fig. 1d, which is herein referred to as the Hierarchical Dog-Bone.

On the other hand, the stress releasing topological designs were inspired by a Japanese paper cutting artform called Kirigami. The Kirigami-based structure allows for enhanced elastic softening and large deformations of an otherwise rigid or non-stretchable substrate material^28^. The Kirigami design shown in Fig. 1e and Supplementary Fig. 1g included a periodic array of horizontal cuts (Supplementary Fig. 1i) that releases stresses when the entire structure is subjected to vertically applied tension. In addition, a Modified Kirigami structure was also introduced (Fig. 1f and Supplementary Fig. 1 h), which has additional curved corner cuts on both ends of the horizontal cuts (Supplementary Fig. 1i) to further release stress concentrations.

### Numerical analysis of stress fields

**Figure 2:**
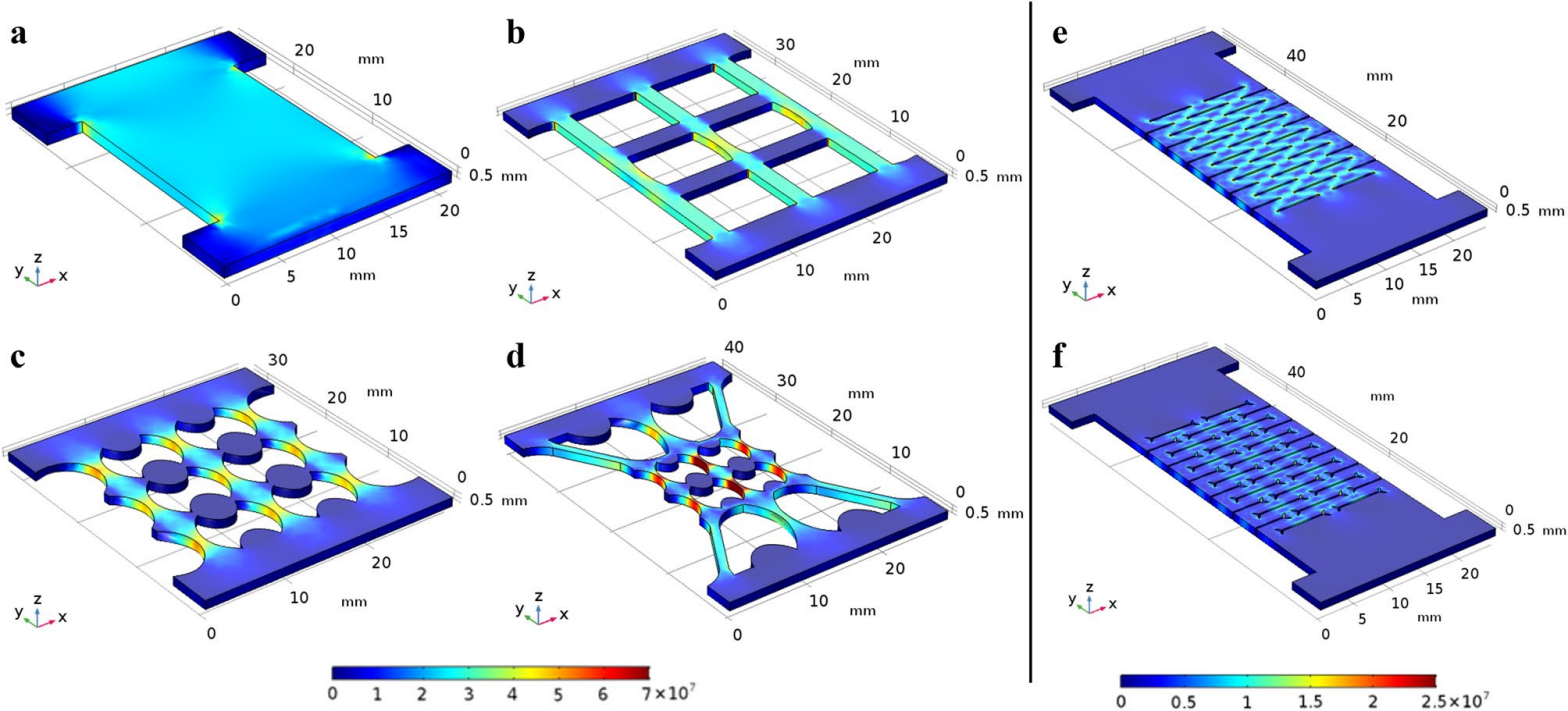
FE analysis of the mechanical response of the different topological designs. Von Mises stress field distributions in the (**a**) Non-patterned, (**b**) Grid, (**c**) Dog-Bone Grid, (**d**) Hierarchical Dog-Bone, (**e**) Kirigami structure, and (**f**) the Modified Kirigami topologies.

FE modeling using the Solid Mechanics Module of *COMSOL Multiphysics* was performed to verify that the various topological designs in Fig. 1 could effectively concentrate or release tension-induced stresses in the films. Figure 2 shows the von Mises stress fields in thin films of different topologies when subjected to a 1% tensile strain applied in the vertical direction. Figure 2a–d indicate that stresses were concentrated in the inhomogeneous vertical elements, and the overall magnitude of stress (in the vertical elements of the dog-bone shape patterns) was increased due to inhomogeneity of the pattern. In addition, the Hierarchical Dog-Bone (Fig. 2d) possessed the most dominant stress concentrations, as well as the highest stress magnitudes in the corresponding inhomogeneous elements. In other words, stress concentrations could be achieved by introducing inhomogeneity in the material topology, and such stress concentrating effects could be enhanced using hierarchical designs.

On the contrary, Kirigami structures were expected to relieve stress concentrations. Figure 2e,f show the stress distributions in the Kirigami and Modified Kirigami designs, respectively. One can see that the stress magnitudes in these topologies were significantly lower than the Non-Patterned control set, as well as versus those of the stress-concentrating topologies. In particular, the Modified Kirigami design (Fig. 2f) was characterized by an even lower stress distribution than the conventional Kirigami structure in Fig. 2e, which was achieved by purposely introducing additional corner cuts at the ends of the horizontal cuts. Therefore, the FE modeling results indicated that the designed hierarchical inhomogeneous topologies led to enhanced stress concentrations, whereas cuts in the film or Kirigami-based topologies effectively reduced stress distribution and stress concentrations.

### Strain sensing characterization of nanocomposite thin films

Nanocomposite thin films of different stress-concentrating and stress-releasing topological designs were experimentally tested to characterize their effects on bulk film piezoresistivity. Two different nanocomposite material systems, namely GNS-EC and CNT-latex, were fabricated, and at least three specimens for each topological design were subjected to strain sensing characterization tests.

**Figure 3:**
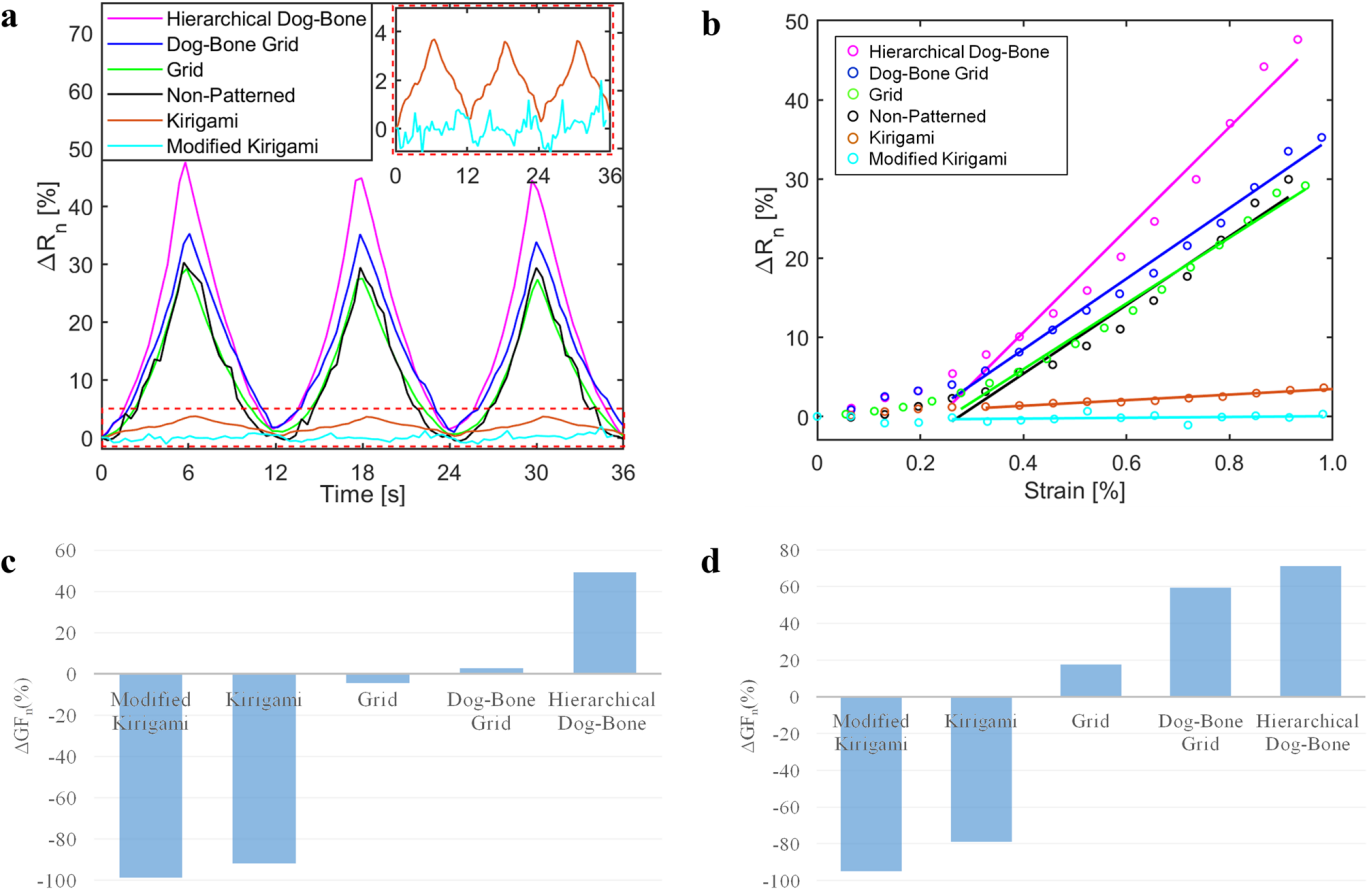
Electromechanical responses of different patterned nanocomposite thin films. (**a**) Representative *ΔR*_*n*_ time histories of the different patterned GNS-EC specimens subjected to the same tensile cyclic strain pattern are overlaid. The inset shows the electromechanical response of the Kirgimi and Modified Kirigami specimens. (**b**) The *ΔR*_*n*_ of the GNS-EC specimens are plotted as functions of the increasingly applied strain during one loading cycle. Linear least-squares regression lines are fitted to data where strain ≥ 0.3%. The *ΔGF*_*n*_ of (**c**) GNS-EC and (**d**) CNT-latex nanocomposite thin films obtained by different topological designs as compared to the Non-patterned control sets, respectively.

Figure 3a shows the representative normalized change in resistance *ΔR*_*n*_ time histories of the control set and patterned GNS-EC thin films when they were subjected to tensile cyclic strains. All the *ΔR*_*n*_ time histories of the Non-Patterned, Grid, Dog-Bone Grid, and Hierarchical Dog-Bone followed closely with the applied tensile cyclic strain pattern in a stable and repeatable manner. In addition, the thin films patterned with stress-concentrating designs exhibited larger normalized changes in resistance (i.e., were more sensitive to strains) than the homogeneous control set.

To better compare the strain sensitivities or gage factors (*GF*) of the different topology GNS-EC nanocomposite thin films, Fig. 3b plots *ΔR*_*n*_ as a function of applied strains (*Δ*ε). Here, *GF* is defined according to Eq. (1):

1 $$GF=\frac{\Delta {R}_{n}}{\Delta \varepsilon }.$$

Although the strain sensing response of the grid structures were polynomial, as is shown in Fig. 3b, linear least-squares best-fit lines were fitted to the data corresponding to ≥ 0.3% applied strains. Then, the slopes of the fitted linear lines were computed as an estimate of thin film *GF*s (according to Eq. 1). It can be seen from Fig. 3b that the linear approximation was able to sufficiently characterize the changing trends of *ΔR*_*n*_ for the various nanocomposite topologies tested. To be specific, the *GF*s of the Grid, Dog-Bone Grid, and Hierarchical Dog-Bone topologies were calculated to be ~ 38, 41, and 60 with errors less than 10%, respectively. This indicated that the bulk film *GF* of the GNS-EC strain sensors could be effectively increased by leveraging the inhomogeneous topology-induced stress concentrations in the material system. In addition, higher levels of hierarchical inhomogeneity led to more significant enhancements in strain sensitivity. These results imply that high-sensitivity sensors could be developed solely based on designing the material’s topology.

It was hypothesized that the piezoresistivity of GNS-EC thin films mainly stem from deformation- and strain-induced disturbances to the percolated and conductive GNS network of the nanocomposite. In particular, applied tensile strains would induce separations between individual or small bundles of GNS to decrease the total number of GNS-to-GNS contacts, thereby reducing the number of overall electrical current conducting pathways in the nanocomposites and thus leading to higher bulk film resistance. Based on this hypothesis, this study focused on manipulating the stress distribution in nanocomposite thin films and used this as a mechanism for controlling their bulk film strain sensitivity. For instance, when higher strain sensitivity is desired, significant disturbances in the GNS-conducting pathways could be achieved by purposefully incorporating stress and strain concentrations in the nanocomposite.

On the other hand, based on the same hypothesis, the Kirigami-based topologies were designed to release stress/strain concentrations in the nanocomposites to reduce disturbances to the percolated GNS networks and to minimize strain sensitivity. From Fig. 3a,b, one can observe that the Kirigami-based nanocomposite specimens exhibited significantly lower strain sensing response. The suppressed strain sensitivity was especially obvious for the Modified Kirigami topology sample set, whose *GF* was found to be ~ 0.48 with 30% errors (Fig. 3b). The relatively higher errors were due to the very low *GF* for the Modified Kirigami design. These results suggested that the global strain sensing performance of piezoresistive nanocomposite thin films could be efficiently suppressed by releasing stresses in the material system and by preserving their nanostructure during large deformations. In other words, the stress-releasing topologies (i.e., Kirigami-based structures) are promising candidates for decoupling sensing signals induced by strains/deformation from the primary desirable measurand.

Overall, Fig. 3c summarizes the normalized difference in *GF*s (*ΔGF*_*n*_ = *(GF*_*i*_* − GF*_*0*_*)/GF*_*0*_) obtained by the proposed topological designs as compared to the Non-Patterned control set for the GNS-EC nanocomposites. Here, *GF*_*i*_ represents the *GF* values of each pattern, while *GF*_*0*_ is that of the Non-Patterned sample set (~ 40). It was found that, based on the same GNS-EC material system, a topological design strategy could achieve a remarkably expanded spectrum (− 99% to + 50%) of strain sensing performance. This indicates that the proposed topological design approach could be potentially leveraged to strategically manipulate and design the bulk material’s piezoresistivity in a predictable and controllable manner.

This study also experimentally characterized the strain sensing performance of patterned CNT-latex nanocomposite thin films to further validate the effectiveness and applicability of this topological design strategy. The representative *ΔR*_*n*_ time histories of the CNT-latex specimens from the strain sensing tests, as well as *ΔR*_*n*_ as a function of *Δ*ε are shown in Supplementary Fig. 2a,b, respectively. In addition, Fig. 3d summarizes the normalized difference in *GF*s (i.e., *ΔGF*_*n*_) obtained by the proposed topological designs as compared to the Non-Patterned control set of CNT-latex specimens. One can observe that the stress-concentrating topologies enhanced the strain sensitivity of the CNT-latex nanocomposites by ~ 70% (i.e., Hierarchical Dog-Bone), while the stress-releasing structures suppressed piezoresistivity by ~ 95% (i.e., Modified Kirigami). These results further demonstrated that the topological design-based approach could consistently manipulate different piezoresistive nanocomposite material systems, paving ways for next-generation multifunctional materials development and strategies for engineering specific material properties.

### Numerical analysis of electromechanical response

While the experimental tests validated tuning of bulk material strain sensitivity, design would require a numerical model that considered the electromechanical properties of the material system. Therefore, two different material models were developed in this work, which included a calibrated linear piezoresistive material model and a percolated inhomogeneous material model (modeling details are described in Supplementary Information). Since the linear piezoresistive material model was unable to simulate the nonlinear behavior observed from experimental data (Fig. 3b and Supplementary Fig. 2b), this section mainly focuses on the performance of the percolated material models.

It was hypothesized that the experimentally observed nanocomposite strain sensing response mainly stemmed from mechanical loading-induced disturbances to its distribution of electrical defects. To be specific, increasingly applied tension could generate more electrical defects in the material system, which would correspondingly increase bulk electrical resistance of the nanocomposite. Such defects were introduced to the percolated inhomogeneous model by seeding the material model with randomly distributed electrical defects (i.e., low electrical conductivity). These randomly distributed inhomogeneous features (i.e., electrical defects) would propagate according to the externally applied mechanical deformations (e.g., tension) and result in an increase in its electrical resistance.

**Figure 4:**
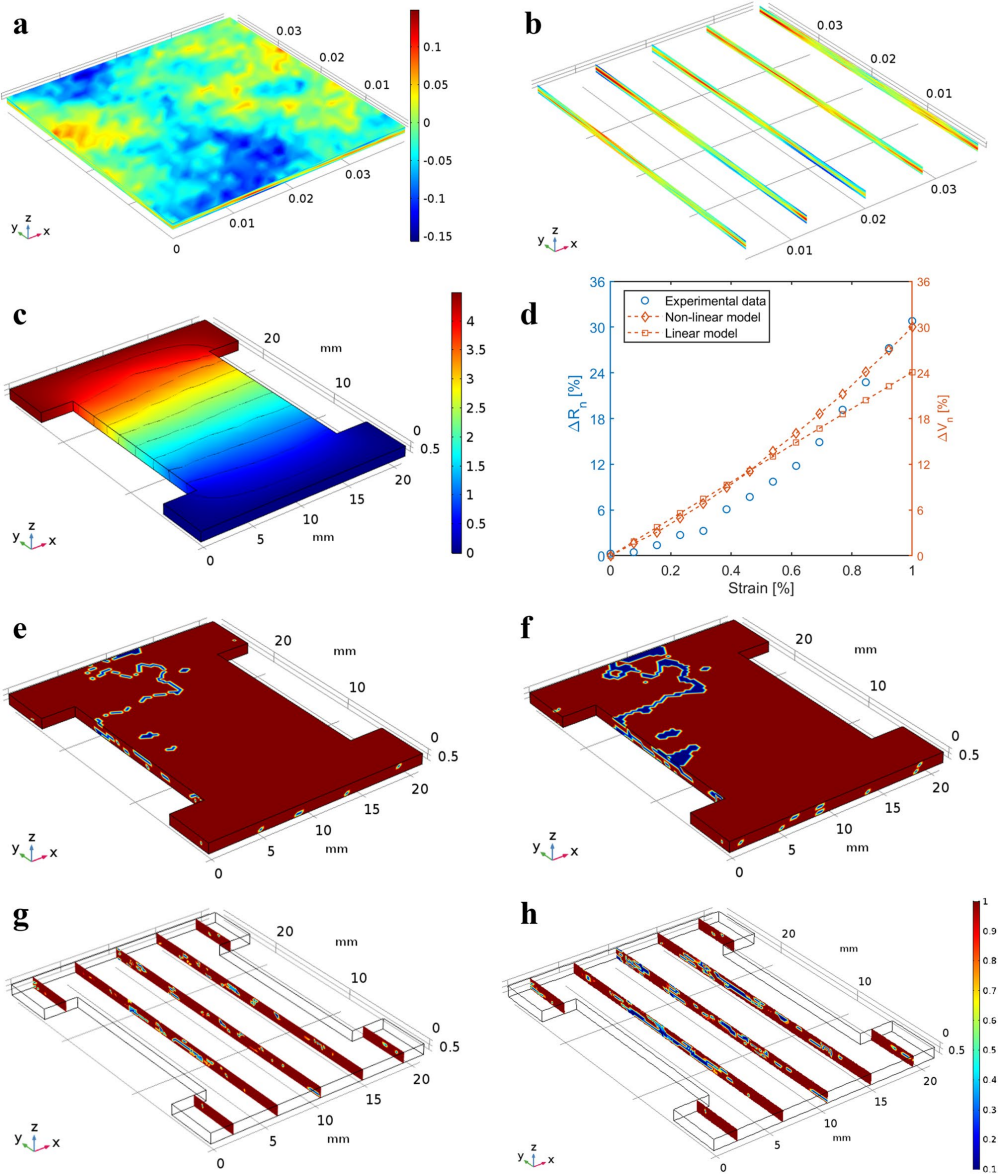
Percolated inhomogeneous material models. (**a**) The synthesized randomized data distribution in the thin slab. (**b**) Randomized data distributions on five slices on the y–z plane within the thin slab. (**c**) The FE model was calibrated using experimental results of the Non-Patterned GNS-EC nanocomposites, and *ΔV*_*n*_ with respect to applied strains are compared to the experimentally measured *ΔR*_*n*_ values. (**d**) The non-linear FE model was calibrated using experimental results from the Non-Patterned topology, and *ΔV*_*n*_ with respect to applied strains are compared to the experimentally measured *ΔR*_*n*_ values as well as that obtained from the linear model. (**e**,**f**) The electrical conductivity distributions in the Non-Patterned material model when it was subjected to 0.5% and 1% tensile strains along the y-axis, respectively. (**g**,**h**) Five cross-sections of the electrical conductivity distributions in (**e**) and (**f**), respectively. (**e**–**h**) Share the same color bar.

Therefore, the percolated inhomogeneous model considered a 3D domain of interest with dimensions of 40 × 40 × 0.1 mm^3^ (i.e., slightly larger than the dimensions of the designed topologies). A randomized statistical dataset was first generated to define the initial set of electrical defects (see Supplementary Information). Figure 4a shows the synthesized random data distributed in the 3D domain, and Fig. 4b shows five slices on the y–z plane of the thin slab to expose the data distribution inside of the slab. The randomized dataset was attributed to each patterned material model by truncating it from the same 3D thin slab.

For the GNS-EC nanocomposite system, the percolated material model was first calibrated based on the Non-Patterned control set. Figure 4c shows the spatial distribution of electric potential, overlapped with isosurfaces of electric potential in the Non-Patterned material model and when it was subjected to 1% tensile strain along the y-axis. The electric potential was nonuniformly distributed, indicating that inhomogeneous electrical conductivity distribution was successfully introduced to the material model. In Fig. 4d, the normalized change in voltage *ΔV*_*n*_ of the calibrated Non-Patterned control set was plotted as a function of applied strains and overlaid with the corresponding experimentally measured *ΔR*_*n*_ results, as well as *ΔV*_*n*_ computed using the linear piezoresistive model, for comparison. Overall, the inhomogeneous material model not only introduced nonlinearity to the simulated strain response but also more accurately characterized the strain sensitivity of the GNS-EC nanocomposites than the linear model. Furthermore, Fig. 4e,f show the electrical conductivity distributions of the calibrated Non-Patterned material model when it was subjected to 0.5% and 1% tensile strains, respectively. Figure 4g,h also show the internal conductivity distributions corresponding to Fig. 4e,f, respectively. The electrical defects clearly propagated in the material when subjected to larger strains.

**Figure 5:**
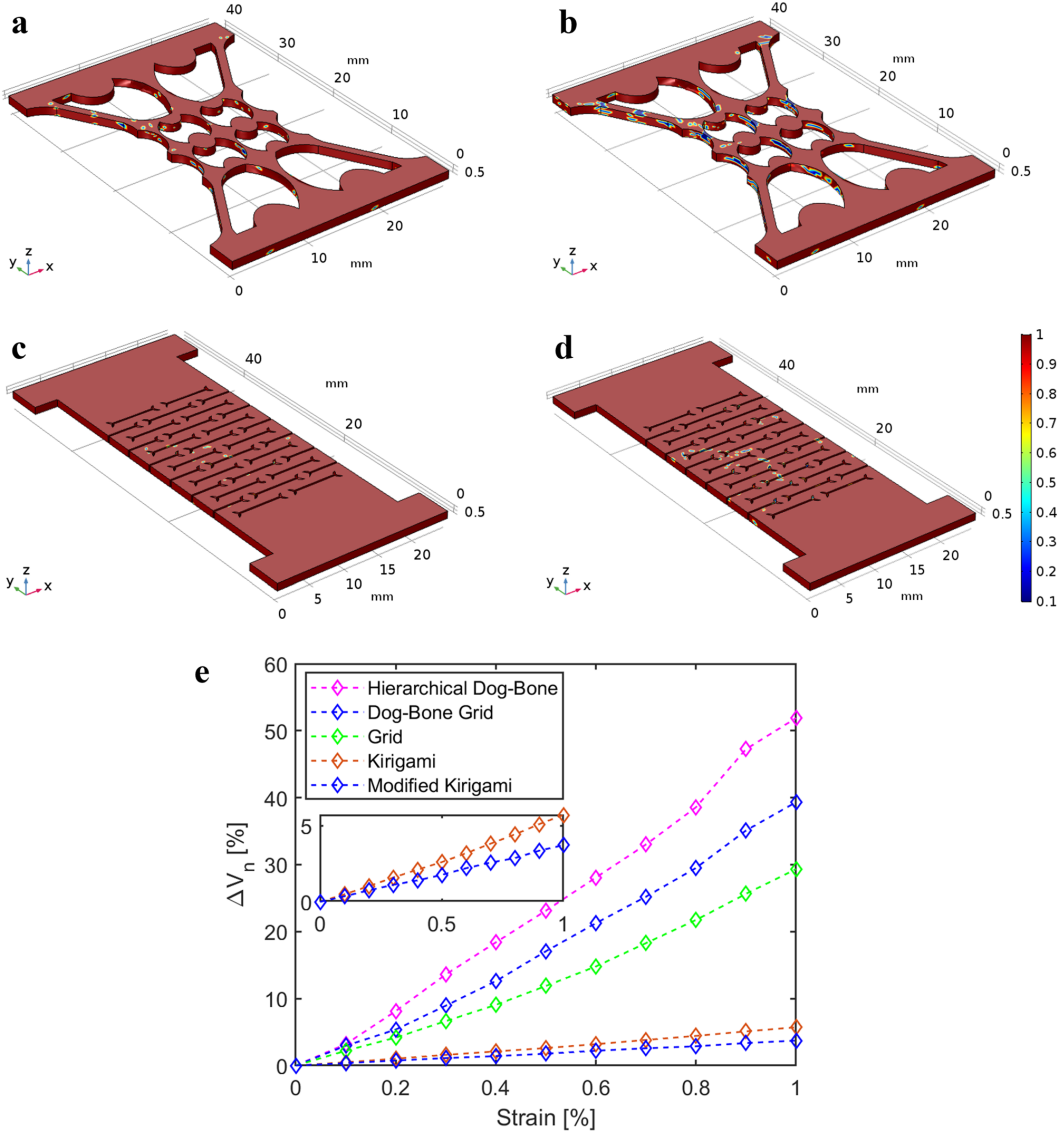
Simulated electromechanical response based on the percolated inhomogeneous material models. (**a**,**b**) Electrical conductivity distributions in the Hierarchical Dog-Bone Grid material model when it was subjected to 0.2% and 1% tensile strains along the y-axis, respectively. (**c**,**d**) Electrical conductivity distributions in the Modified Kirigami material model when it was subjected to 0.2% and 1% tensile strains along the y-axis, respectively. (**a**–**d**) Share the same color bar. (**e**) Simulated electromechanical responses of different topological material models when they were subjected to up to 1% tensile strain. The inset shows a zoomed-in view of the Kirigami-based material models’ electromechanical responses.

Then, the calibrated material model was implemented to simulate the electromechanical responses of the other patterned material models. Figure 5a–d demonstrate the electrical defects distributions and development in the Hierarchical Dog-Bone and Modified Kirigami material models when they were subjected to 0.2% and 1% strains, respectively. The electrical defect distributions of the Grid, Dog-Bone Grid, and Kirigami are shown in Supplementary Figs. 4–6, respectively. Based on Fig. 5a–d and Supplementary Figs. 4–6, it can be observed that electrical defects mainly formed and propagated at the stress-concentrating regions. For the Kirigami topologies, since stress was effectively released from the material, the electrical defects barely developed even at 1% strain. In addition, Fig. 5e overlays the simulated electromechanical responses of all the patterned material models as functions of applied strains. The proposed inhomogeneous material models agreed well with the experimental strain sensing test results, where both showed that the stress-concentrating topologies could enhance nanocomposites thin film piezoresistivity, while the stress-releasing topologies could significantly suppress their strain sensing responses.

## Conclusions

This paper focused on investigating the effects of topological designs on the piezoresistive performance of nanocomposite thin films. The hypothesis was that the strain sensitivity of a piezoresistive nanocomposite could be controlled by tailoring their topologies and their corresponding stress and strain distribution under applied loading. To engineer and control stress distributions in these thin films, two types of topologies were investigated, namely, stress-concentrating patterns and Kirigami-based stress-releasing structures. FE models of these topologies simulated the stress distributions in the patterned thin films and showed that they could effectively change thin film mechanical response. Then, patterned GNS-EC and CNT-latex nanocomposites were fabricated, whose strain sensing performance was characterized through electromechanical tests. The test results showed that, regardless of the nanocomposite material system considered, stress-concentrating topologies enhanced bulk film strain sensitivity, while the Kirigami-based stress-releasing topologies effectively suppressed their piezoresistivity. To facilitate their design and tuning of strain sensing properties, a linear piezoresistive material model and an inhomogeneous percolated material model were developed and implemented to simulate nanocomposite electromechanical properties. It was found that both models suggested similar topological effects on the nanocomposites’ piezoresistive behavior as was observed in the experiments. However, the statistical randomized data-based percolated material model could more accurately characterize the nonlinear strain sensing response of the nanocomposite thin films. Overall, this work demonstrated that the topological design-based approach holds remarkable promise for strategically engineering the performance and properties of functional materials for applications such as wearable thin film sensors. Stress-concentrating topologies can be employed to measure skin-strains more precisely during functional movements, while stress-releasing topologies on the same wearable sensor can be leveraged for placing integrated circuits and microchips (which cannot sustain large strains) for data acquisition and wireless communication purposes. In addition, the model-based design methodology could potentially overcome current empirical material development strategies while efficiently encoding predictable material performance and multifunctionalities during manufacturing.

## Methods

### Finite element analysis of stress field

FE modeling using the Solid Mechanics Module of *COMSOL Multiphysics* was performed to simulate the stress distribution of the various topological designs considered. *AutoCAD* drawings of different topological designs were imported to *COMSOL* to build the model geometry; thickness was manually assigned to be 1 mm. The material’s mechanical properties were assumed to be dominated by the nanocomposite’s substrate, which was polyethylene terephthalate (PET) and assumed to be linear-elastic (i.e., Young’s modulus: 3.5 GPa; Poisson’s ratio: 0.39; density: 1300 kg m^−3^). One end of the material model was fixed, while the other end was assigned a 1% tensile strain applied along the y-axis or vertical direction.

### Fabrication of patterned nanocomposite thin films

#### Materials

The CNTs used in this study were multi-walled and were purchased from NanoIntegris (whose outer diameter is ~ 10 to 20 nm, and purity exceeds 95%). Latex solution was from Kynar Aquatec. Poly(sodium 4-styrenesulfonate) (PSS) (molecular weight is ~ 1 M) and *N*-methyl-2-pyrrolindinone (NMP) were acquired from Sigma–Aldrich. Low-defect few-layer GNS were synthesized from graphite microcrystalline powders (− 325 mesh, 99.995% pure, Alfa Aesar) using a surfactant-free, efficient, and economical LPE process^8,29^ by using a water-NMP (99% extra pure, Acors Organics) mixed solvent. EC was purchased from Sigma-Aldrich (viscosity 100 cP, 5% in toluene/ethanol 80:20, 48% ethoxyl). Conductive silver paint was purchased from Ted Pella for establishing electrodes on the fabricated nanocomposites. The substrates used were PET sheets of ~ 100 µm in thickness. Other chemicals and disposable laboratory supplies were from Fisher-Scientific.

#### Screen printing of GNS-EC nanocomposites

The schematics of the fabrication procedures are shown in Supplementary Fig. 7. First, the dispersing agent was prepared by dissolving EC in ethanol through 24 h of stirring at room temperature. Then, 2 mg mL^−1^ GNS were mixed with the EC/ethanol solution, and the mixture was subjected to 2 h of ice bath sonication (Supplementary Fig. 7a). Here, the ice bath could effectively minimize ethanol evaporation, which would otherwise induce GNS agglomerations. To achieve optimal solution viscosity for screen printing, the dispersed GNS-EC/ethanol solution was heated with a hot plate at 50 °C for ~ 10 min (Supplementary Fig. 7b)^9^. It should be noted that the solution was continuously stirred during heating to guarantee uniform heating and efficient evaporation of ethanol. After obtaining the viscous GNS-based solution, it was coated onto PET substrates through masks, whose patterns could be pre-cut using a laser cutter (Supplementary Fig. 7c). In this study, consistent fabrication of all topology designs was performed by laser-cutting PET substrates (thickness: ~ 100 µm) using a 40 W CO_2_ benchtop laser cutter (Orion MotorTech). Digital *AutoCAD* drawings were uploaded, and the laser-cutter faithfully reproduced these patterns in the PET. Finally, the coated GNS-EC patterns were air-dried overnight at room temperature to evaporate any residual ethanol. Electrodes were established at opposite ends of the nanocomposite for facilitating electrical characterization testing of GNS-EC nanocomposites, as shown in Supplementary Fig. 7d.

#### Spray coating of CNT nanocomposites

CNT-based nanocomposite thin films were fabricated and utilized as the strain sensing elements^30,31^. The CNT-latex thin films were sprayed following the steps described in Refs.^31,32^. In short, CNTs were added to a 2 wt% PSS aqueous solution with dilute amounts of NMP. The CNT-surfactant mixture was then subjected to high-energy probe ultrasonication (3 mm tip, 150 W, 22 kHz) for 1 h to fully disperse the CNTs, after which a Kynar Aquatec latex solution and deionized (DI) water were added to obtain the final sprayable ink. Finally, the as-prepared ink was spray coated onto the pre-patterned PET substrates using an airbrush and air-dried in ambient room temperature. The patterned CNT-latex nanocomposites are shown in Supplementary Fig. 8.

### Strain sensing tests

To prepare patterned nanocomposite thin films for strain sensing tests, colloidal silver paste and copper tape electrodes were established at opposite ends of each film for two-point probe electrical resistance measurements. Then, the strain sensing response of the patterned nanocomposite thin films was experimentally characterized by conducting load tests using a Test Resources 150R load frame. Here, each specimen was subjected to uniaxial tensile cyclic strains (load rate: 10%/min; peak strain: 1%), while a Keysight 34465A DMM simultaneously measured the bulk film resistance at a sampling rate of 2 Hz. Data was recorded using a Keysight *BenchVue* software.

## Supplementary Information

Supplementary Information.
