# Supplementary material for: Topological design of strain sensing nanocomposites
Source: Sci Rep. 2022 Jun 2;12:9179. doi: 10.1038/s41598-022-13393-w (PMC9163329; doi:10.1038/s41598-022-13393-w)
Supplement: Supplementary file 1 — Supplementary Information. [file 41598_2022_13393_MOESM1_ESM.docx]

**Supplementary Information**

**Topological Design of Strain Sensing Nanocomposites**

# Long Wang^1,2^, Wei-Hung Chiang^3^, and Kenneth J. Loh^1,^*

^1^Department of Structural Engineering, University of California San Diego, La Jolla, CA, 92093-0085, USA

^2^Department of Civil and Environmental Engineering, California Polytechnic State University, San Luis Obispo, CA, 93407, USA

^3^Department of Chemical Engineering, National Taiwan University of Science & Technology, Taipei 106, Taiwan

*E-mail: kenloh@ucsd.edu

# Supplementary Note 1

**Linear piezoresistive material models**

Finite element numerical simulations were performed using COMSOL by coupling the Solid Mechanics Module with the Electric Currents Module. Initially, the nanocomposite thin films were simulated by modifying the material properties of the COMSOL built-in material model for piezoresistive p-doped silicon. The piezoresistive coupling matrix (𝛱) was calibrated to fit the experimental measurements for the control set of Non-Patterned specimens, including both GNS-EC and CNT-latex nanocomposites for the corresponding material systems. In general, for piezoresistive materials, the current electric field strength (*E*) can be expressed as follows:

$E= \rho\cdot I+\Delta\rho\cdot I$ (1)

where *I* represents current, *ρ* is the original resistivity of the material, and *∆ρ* is the change in resistivity resulting from mechanical excitations (*e.g.*, applied loads). In other words, *∆ρ* is related to mechanical stress (*σ*), which can be expressed in the form of constitutive relationship, as follows:

$\Delta\rho= \Pi\cdot\sigma$ (2)

In the context of three-dimensional (3D) modeling of cubic symmetry materials with isotropic conductivity (*e.g.*, silicone), Equation 3 becomes:

$\left[ \begin{matrix} \Delta\rho_{xx} \\ \Delta\rho_{yy} \\ \Delta\rho_{zz} \\ \Delta\rho_{yz} \\ \Delta\rho_{xz} \\ \Delta\rho_{xy} \end{matrix} \right]=\left[ \begin{matrix} \pi_{11} & \pi_{12} & \pi_{12} & 0 & 0 & 0 \\ \pi_{12} & \pi_{11} & \pi_{12} & 0 & 0 & 0 \\ \pi_{12} & \pi_{12} & \pi_{11} & 0 & 0 & 0 \\ 0 & 0 & 0 & \pi_{44} & 0 & 0 \\ 0 & 0 & 0 & 0 & \pi_{44} & 0 \\ 0 & 0 & 0 & 0 & 0 & \pi_{44} \end{matrix} \right]\cdot\rho\left[ \begin{matrix} \sigma_{xx} \\ \sigma_{yy} \\ \sigma_{zz} \\ \sigma_{yz} \\ \sigma_{xz} \\ \sigma_{xy} \end{matrix} \right]$ (3)

where 𝜋*_ij_* are the elements of the piezoresistive coupling matrix 𝛱 and are defined as piezoresistive coefficients. Assuming the nanocomposites also possess similar isotropic electrical properties as silicone, the piezoresistive coefficients of the nanocomposite material models were first linearly calibrated based on the experimentally measured strain response (*i.e.*, *ΔR_n_* time histories) of the Non-Patterned sample set. Then, the calibrated material models were used to simulate the electromechanical responses of different topological designs, whose results were compared with the corresponding experimental measurements. Here, the fixed-end of each material model was assigned as electrical ground, while the other end (*i.e.*, where tension was applied) was set as the terminal. A direct current of 2 mA was then injected between the ground and terminal, which was similar to the experimental resistance measurement mode of the digital multimeter used.

First, for the GNS-EC nanocomposite thin films, Supplementary Fig. 3a shows the spatial distribution of electric potential, as well as isosurfaces of electric potential in the Non-Patterned material model, when it was subjected to 1% tensile strain applied in the direction of the y-axis. Its electric potential is uniformly distributed in the material except for regions near the boundaries (due to ground and terminal effects), which was consistent with the assumption of homogeneous material properties. In addition, the strain response of the material models could be obtained by probing the terminal electric potential for each deformation case. In Supplementary Fig. 3b, the normalized change in electric potential (*ΔV_n_=ΔV/V_0_*) of the calibrated Non-Patterned control set was plotted as a function of applied strains and overlaid with the corresponding experimentally measured *ΔR_n_* results. Here, *ΔV* and *V_0_* represent the change in the terminal electric potential and initial (unstrained) terminal electric potential, respectively. It can be seen that the simulated material possessed similar strain sensitivity as the actual GNS-EC nanocomposite thin films.

The calibrated material model’s piezoresistivity was then directly assigned to the different topological models for characterizing their electromechanical performance. Supplementary Fig. 3c shows the variations of *ΔV_n_* for different material model topologies when subjected to increasingly applied tensile strains up to 1%. Similar to the experimental results, one can find that the simulated stress-concentrating topologies were more sensitive to strain, while the stress-releasing ones could decrease strain sensitivity. In the meantime, it should be noted that the linear numerical model underestimated the absolute values of the strain sensing responses for the corresponding specimens. In addition, this study also implemented the same FE analysis approach to simulate the electromechanical responses of the CNT-latex nanocomposite thin films. Supplementary Fig. 3d shows the computed *ΔV_n_* for different topological material models, including the pre-calibrated Non-Patterned control set. The linear piezoresistive model could also simulate the relative change in strain sensitivity introduced by different topological designs. Overall, the linear electromechanical FE analyses successfully demonstrated how thin film topologies would affect bulk film strain sensitivity, and the results were consistent with those observed from the experiments.

# Supplementary Note 2

**Percolated inhomogeneous material models**

The experimentally measured strain sensing response exhibited slight nonlinearities that could not be simulated using the linear piezoresistive numerical model. It is hypothesized that the nonlinear strain response potentially resulted from inhomogeneous changes in the nanocomposites’ microstructure during applied strains and deformations^1,2^. Thus, to further enhance the accuracy of the electromechanical numerical simulations, another approach was considered to account for potential inhomogeneous material property changes using nonlinear percolated material models.

First, to generate a randomized statistical data set, a combination of trigonometric functions was used as follows:

$f(x,y,z)= \sum_{k=-K}^{K} \sum_{l=-L}^{L} \sum_{m=-M}^{M} a(k,l,m)cos(2N\pi(kx+ly+m(40z))+\varphi(k,l,m))$ (4)

where the slowest oscillation of the cosine waves was defined by *2𝜋/2N𝜋* *= 1/N*, which determined the periodicity of the synthesized data. With *N* = 25 used in this study, the spatial period length was 40 mm so that the 3D domain of interest could include all the randomly generated dataset without bias. On the other hand, *k*, *l*, and *m* are spatial frequencies that determined the fastest oscillations of the cosine waves, whose maximum values are *K*, *L*, and *M*, respectively. Generally, larger spatial frequencies could improve the spatial resolution of the data, but it would also increase computational cost, especially for 3D cases. In this study, considering the dimensions of the material models, *K = L = M = 200* m^-1^; then the shortest wavelengths in the x, y, and z directions were 0.2 mm. To avoid generating cosine wave functions of biased oscillation directions, *k*, *l*, and *m* were allowed to take on negative values. In addition, *φ(k, l, m)* represents phase angles, which were generated with a uniform distribution that ranged from *-𝜋/2* to *𝜋/2*. Furthermore, $a(k,l,m)$represents oscillation amplitude coefficients, which were determined by:

$a(k,l,m) = \frac{g(k,l,m)}{{(k^{2}+l^{2}+m^{2})}^{\frac{\beta}{2}}}$ (5)

where the function $g(k,l,m)$had a random Gaussian distribution ($g(k,l,m)$ *~* $N(0, 1)$), and *β* is the spectral exponent to attenuate higher frequencies so as to generate smooth amplitude coefficients. Since the 3D domain is a thin slab (*i.e.*, smaller z-dimensions), the z-coordinate values were scaled up 40 times to include the entire dataset. The synthesized random data distribution is shown in Figs. 5a and 5b. At this point, the randomized data were dimensionless and did not represent any physical properties.

Then, to employ the dimensionless synthesized data to represent physical property parameters, one could multiply Equation 4 with a corresponding function that holds corresponding physical meaning. To use the randomized statistical dataset to represent material electrical conductivity distribution in this work, Equation 4 was multiplied by a simplified constant *C* [S m^-1^], which ideally should be calibrated with experimental data (*i.e.*, nanocomposite conductivity measurements). However, since this study would mainly evaluate the normalized changes in the terminal electrical potential of the material models under tension, as opposed to absolute values of the electrical potentials, obtaining the exact value of *C* was not crucial. Furthermore, in order to introduce the aforementioned electrical defects (*i.e.*, inhomogeneous material features), a Boolean expression was implemented to convert the randomized conductivity values into binary data distribution, and it can be expressed as follows:

$\sigma(x,y,z)=C[0.1+0.9\times(|f(x,y,z)|>\tau(x,y,z))]$ (6)

where *σ(x, y, z)* represent the conductivity value at coordinate (*x, y, z*), and *τ(x, y, z)* is a pre-defined threshold function. Here, the absolute values of the synthesized randomized data were considered for mathematical simplicity. Equation 6 indicates that the electrical conductivity at a specific location could only be either 0.1*C* or *C*, which represents electrical defects and intact electrical connection, respectively.

In addition, due to the experimentally observed nanocomposite thin film piezoresistivity, their electrical conductivity should be coupled with their mechanical response when subjected to applied tensions. Based on the previously mentioned hypothesis, electromechanical performance coupling was obtained by establishing the following relationship:

$\tau(x,y,z)=\phi(\frac{\gamma_{i}(x,y,z)}{\gamma_{0}})$ (7)

where *γ_i_(x, y, z)* represents the von Mises stress values at coordinate (*x, y, z*) when the patterned material model was subjected to uniaxial tensile strains (ranging from 0 to 1%). *γ_o_* is the von Mises stress value at the center location of the Non-Patterned material model under 1% tensile strain (*i.e.*, control stress). Both *γ_i_(x, y, z)* and *γ_0_* could be obtained from FE analyses of the stress distributions of different material models. In other words, Equation 7 evaluates the relative changes in stress distribution introduced by the designed topologies, as compared to the Non-Patterned control set. In addition, the *ϕ* function could be established by calibrating the percolated material model for the control set with corresponding experimental data. The simplest form of the *ϕ* function would be a linear function, whose simulated responses for the patterned material models were found to be more accurate than those obtained from nonlinear *ϕ* functions. This was because the patterned material models featured much higher stresses than the Non-Patterned control set under the same number of deformations (*i.e.*, large $\frac{\gamma_{i}(x,y,z)}{\gamma_{0}}$ values), and the nonlinear *ϕ* functions performed poorly during extrapolation. Thus, the remainder of this study used a monotonically increasing linear *ϕ* function. From Equations 6 and 7, one can estimate that more concentrated stresses would lead to more electrical defects, whereas released stress would avoid electrical defect formation and propagation, which was consistent with the initial hypothesis. Supplementary Figs. 4 to 6 show the electrical defects distributions and development in the Grid, Dog-Bone Grid, and Kirigami material models when they were subjected to 0.2% and 1% strains, respectively.

|  |
| --- |
| **Supplementary Figure 1.** The AutoCAD drawing details for different topological designs. **a** Grid, **b** Dog-Bone Grid (whose detailed dimensions shown in **c**, and **d** Hierarchical Dog-Bone (whose detailed dimensions shown in **e** and **f**) topologies. **g** Kirigami and Modified Kirigami designs (whose periodic units dimensions are shown in **i** corresponding to the highlighted regions in **g**). |

|  |
| --- |
| **Supplementary Figure 2.** Strain sensing responses of different patterned CNT-latex thin films. **a** Representative *ΔR_n_* time histories of the different patterned CNT-latex specimens subjected to the same tensile cyclic strain pattern are overlaid. **b** The *ΔR_n_* of the CNT-latex specimens are plotted as functions of the increasingly applied strain during one loading cycle. Linear least-squares regression lines are fitted to data where strain ≥ 0.3%. |

|  |
| --- |
| **Supplementary Figure 3.** Simulated electromechanical responses based on the linear piezoresistive material models. **a** The electrical potential distribution along with the isosurfaces of electric potential in the Non-Patterned material model when subjected to 1% tensile strain is plotted. **b** The FE model was calibrated using experimental results of the Non-Patterned GNS-EC nanocomposites, and *ΔV_n_* with respect to applied strains are compared to the experimentally measured *ΔR_n_* values. The simulated electromechanical responses of **c** GNS-EC and **d** CNT-latex nanocomposites when they were subjected to up to 1% tensile strain, respectively. The insets show a zoomed-in view of the Kirigami-based material models’ electromechanical responses. |

|  |
| --- |
| **Supplementary Figure 4.** Simulated inhomogeneous electrical conductivity distributions based on the percolated material models. **a – b** The electrical conductivity distributions in the Grid material model when it was subjected to 0.2% and 1% tensile strains along the y-axis, respectively. **c – d** Five cross-sections of the electrical conductivity distributions in **a** and **b**, respectively. **a – d** share the same color bar. |

|  |
| --- |
| **Supplementary Figure 5.** Simulated inhomogeneous electrical conductivity distributions based on the percolated material models. **a – b** The electrical conductivity distributions in the Dog-Bone Grid material model when it was subjected to 0.2% and 1% tensile strains along the y-axis, respectively. **c – d** Five cross-sections of the electrical conductivity distributions in **a** and **b**, respectively. **a – d** share the same color bar. |

|  |
| --- |
| **Supplementary Figure 6.** Simulated inhomogeneous electrical conductivity distributions based on the percolated material models. **a – b** The electrical conductivity distributions in the Kirigami material model when it was subjected to 0.2% and 1% tensile strains along the y-axis, respectively. **c – d** Five cross-sections of the electrical conductivity distributions in **a** and **b**, respectively. **a – d** share the same color bar. |

| 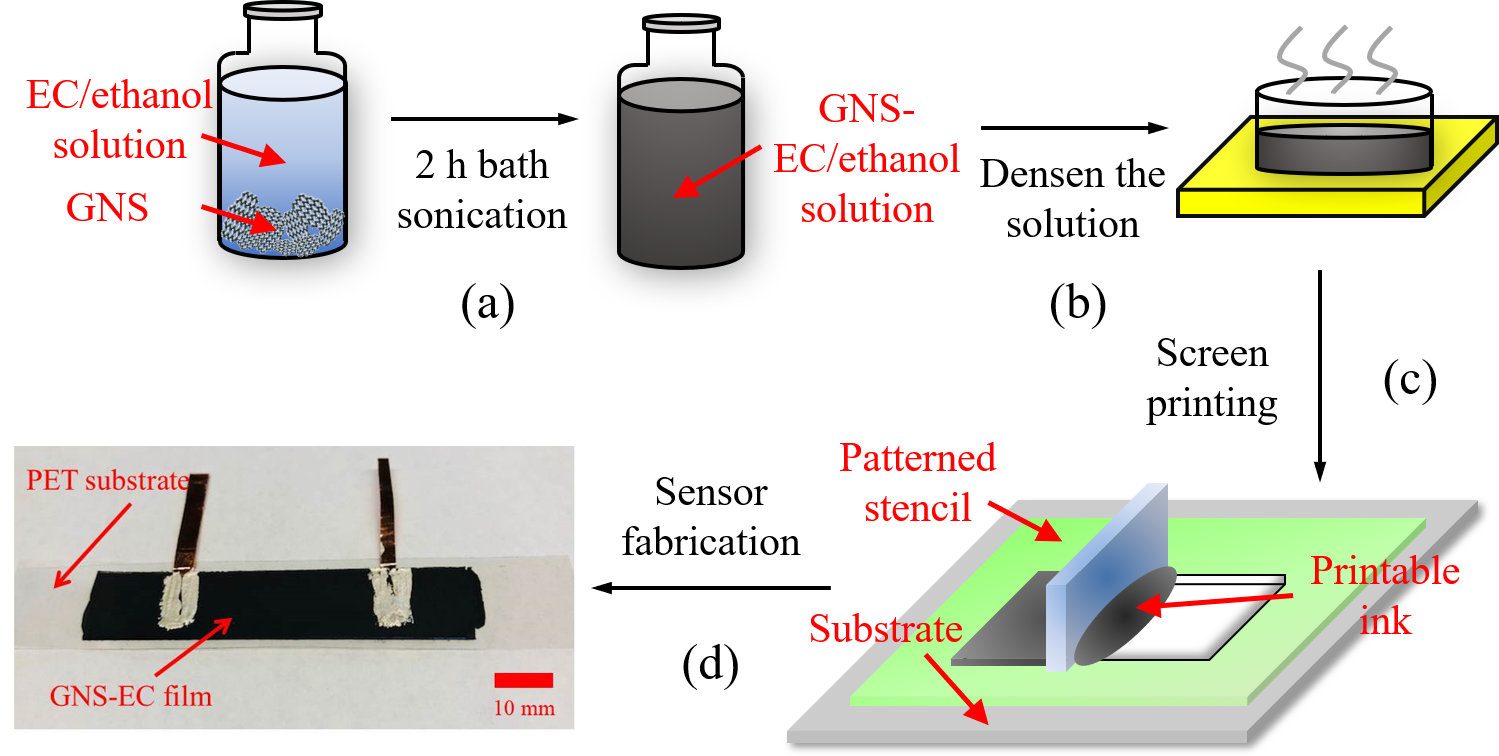 |
| --- |
| **Supplementary Figure 7.** Schematics of the fabrication procedures of GNS-EC nanocomposite thin films. **a** Disperse GNS in the EC-ethanol solution using sonication. **b** Thermal treatment of the dispersed solution to evaporate ethanol. **c** Screen printing the ink over patterned masks onto the substrates. **d** Establishing copper tape electrodes on the GNS-EC thin films for strain sensing tests. |

|  |
| --- |
| **Supplementary Figure 8.** Images of CNT-latex nanocomposites patterned into **a** Grid, **b** Dog-Bone Grid, **c** Hierarchical Dog-Bone, **d** Kirigami, and **e** Modified Kirigami topologies. |

**References**

1 Amjadi, M., Pichitpajongkit, A., Lee, S., Ryu, S. & Park, I. Highly stretchable and sensitive strain sensor based on silver nanowire–elastomer nanocomposite. *ACS nano* **8**, 5154-5163 (2014).

2 Amjadi, M., Kyung, K. U., Park, I. & Sitti, M. Stretchable, Skin‐Mountable, and Wearable Strain Sensors and Their Potential Applications: A Review. *Advanced Functional Materials* **26**, 1678-1698 (2016).
